# CARING FOR OLDER ADULTS ACROSS THE CARE CONTINUUM: EXPERIENCES OF OLDER ADULTS AND FORMAL CAREGIVERS

**DOI:** 10.1093/geroni/igac059.1618

**Published:** 2022-12-20

**Authors:** Rose Ann DiMaria-Ghalili, Rose Ann DiMaria-Ghalili

## Abstract

As individuals age, they are at risk of experiencing health challenges that can contribute to poorer health outcomes and increase health care costs. During acute care crises and through the continuum of dealing with chronic diseases, older adults rely on formal caregivers to provide care and support. Formal caregivers’ delivery of care can be challenged during difficult patient cases (e.g., providing wound care to a person exhibiting behavioral and psychological symptoms of dementia) and during high stress times, such as working through a pandemic. However, during difficult times, resilience among formal caregivers can have a positive affect on care delivery. This symposium identifies some of the health challenges experienced by aging adults, challenging experiences of nurses, levels of resilience among formal caregivers, and discusses the implications for nursing care and future research. Ms. Coates examines trends in adverse drug admissions among older adults in the United States using the 2018 Healthcare Cost and Utilization Project’s National Inpatient Sample. Dr. Hwang explores the relationship between nutrition, inflammation, and physical and mental health in aging adults with chronic leg wounds. Dr. Sefcik discusses nurses’ experiences caring for community-dwelling persons living with dementia and chronic wounds. Mr. Hathaway examines factors that contribute to resilience in staff of a continuing care retirement community during the late stage of the COVID-19 pandemic. Dr. DiMaria-Ghalili, discussant, will synthesize these findings and offer insights on designing interventions to address health challenges and recommendations for supporting formal caregivers.

